# Visual discrepancies in the identification of *Haemophilus influenzae* through gram staining blood culture and sputum smears

**DOI:** 10.1016/j.idcr.2023.e01838

**Published:** 2023-07-02

**Authors:** Naoya Itoh, Nana Akazawa, Makoto Yamaguchi, Yohei Kobayashi, Shinichiro Shibata

**Affiliations:** a Division of Infectious Diseases, Aichi Cancer Center Hospital, 1-1 Kanokoden, Chikusa-ku, Nagoya, Aichi 464-8681, Japan; b Collaborative Chairs Emerging and Reemerging Infectious Diseases, National Center for Global Health and Medicine, Graduate School of Medicine, Tohoku University, 2-1 Seiryo-machi, Aoba-ku, Sendai, Miyagi 980-8575, Japan; c Microbiology Department, Nagoya City Public Health Research Institute, Nagoya, Aichi, Japan

A 67-year-old Japanese man with advanced hypopharyngeal cancer presented with fever, chills, cough, and sputum. Approximately six years ago, the patient was diagnosed with inoperable hypopharyngeal cancer approximately and was treated with chemotherapy and radiation therapy. Five days prior to admission, the patient presented with persistent fever, cough, and sputum. Chest radiography revealed infiltration in the left middle and lower lung fields, and was admitted for aspiration pneumonia. Gram staining of the sputum on the day of admission showed polymorphic gram-negative bacilli, gram-positive bacilli, and gram-positive cocci (Fig. 1A). The patient was administered ampicillin-sulbactam (ABPC/SBT) (12 g/day) empirically. On the day after admission, one blood culture showed long, elongated gram-negative bacilli in anaerobic bottle (Fig. 1B). The discrepancy between the results of sputum and blood culture smears for Gram-negative bacteria was concerning. However, assuming the presence of anaerobic gram-negative rods or β-lactamase-negative ampicillin-resistant (BLNAR) *H. influenzae*, we increased the ABPC/SBT regimen with intravenous levofloxacin (500 mg/day). Gram-negative rods were poorly developed in subcultures of blood and sputum cultures, and could not be identified by Vitek2 (bioMérieux Japan Co., Ltd., Tokyo, Japan). Matrix-assisted laser desorption/ionization time-of-flight mass spectrometry using the Vitek MS ver. 3.2 system (bioMérieux Japan Co., Ltd.,) demonstrated the presence of *H. influenzae* with a 99.9% confidence interval. Moreover, *Stenotrophomonas maltophilia*, α-haemolytic Streptococcus, *Corynebacterium* species, and *Candida albicans* were isolated from sputum cultures. The final diagnosis was an invasive *H. influenzae* infection. Antimicrobial susceptibility testing revealed that *H. influenzae* was BLNAR. The blood and sputum *H. influenzae* isolate was sent to the Nagoya City Public Health Research Institute for typing and was found to be non-typable by gene capsule analysis. The fever resolved on the sixth day of hospitalization, and the cough and sputum production improved. ABPC/SBT and LVFX were intravenously administered for 10 days, followed by oral LVFX 500 mg/day for 4 days.

**Fig. 1:**
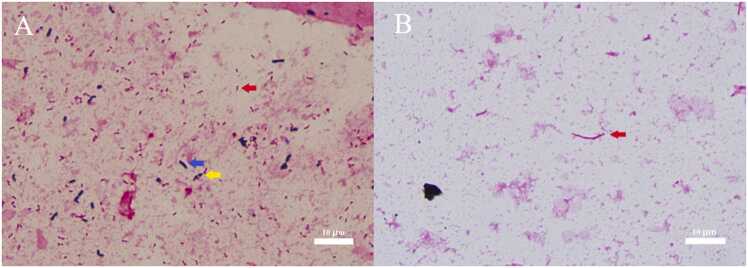
(A) Gram staining of sputum. (B) Gram staining of blood culture.

While *H. influenzae* is typically characterized as small, pleomorphic, Gram-negative bacilli, the blood culture from this case displayed an atypically different morphology from that of the sputum [1]. To the best of our knowledge, there are no reports on Gram staining findings of *H. influenzae* in blood cultures. The forms of *H. influenzae* in the blood and sputum smears may vary based on the environmental conditions. Given that invasive *H. influenzae* infections are rare, that can occasionally be fatal [2], our findings hold valuable potential for facilitating rapid diagnosis and treatment.

## Ethical approval

Not applicable.

## Data Availability

All the relevant data are contained in the report.

## Funding

This research did not receive any specific grant from funding agencies in the public, commercial, or not-for-profit sectors.

## Declaration of interest

None.
